# Investigating acceptability of a training programme in precision medicine for frontline healthcare professionals: a mixed methods study

**DOI:** 10.1186/s12909-022-03613-2

**Published:** 2022-07-19

**Authors:** Sharon Mitchell, Evrim Jaccard, Felix Michael Schmitz, Elianne von Känel, Prune Collombet, Jacques Cornuz, Gérard Waeber, Idris Guessous, Sissel Guttormsen

**Affiliations:** 1 Institute of Medical Education (IML), University of Bern, 3201 Bern, Switzerland; 2 Department of Medicine, University Hospital CHUV, Lausanne, 1011 CH Switzerland; 3 Institute of Psychology, University of Bern, Fabrikstrasse 8, Bern, 3012 CH Switzerland; 4 Primary Care Medicine, Faculty of Medicine, Geneva University Hospital (HUG), Geneva, 1205 CH Switzerland; 5 Faculty of Biology and Medicine, Unisanté, University of Lausanne, Lausanne, 1011 CH Switzerland

**Keywords:** Precision medicine, Continuing professional development, Conceptual frameworks, Implementation Science outcomes

## Abstract

**Background:**

Precision Medicine offers tailored prevention, diagnosis, treatment and management to patients that considers genomics, lifestyle and environmental factors. If implementation of Precision Medicine is to advance, effective, focused upskilling of frontline healthcare professionals through quality continuing professional development is needed. This study reports on an evidence-based approach to needs assessment to investigate the current level of knowledge of Precision Medicine, acceptable content for training, the perceived potential of a more precision approach to patient care and motivation to participate in a training programme among pharmacists, advanced practice nurses and general practitioners. Investigating perceived needs can avoid a top-down approach and support a design that is fit for purpose to targeted professions.

**Methods:**

This study reports on 2 focus groups (*n* = 12) delivered in French and German with equal professional participation of the targeted professions. The research objectives were investigated in two phases. During the first phase, a literature review and expert consultations were conducted to develop a definition of PM, patient cases and content for training. In a second phase, these investigations were further explored using focus groups to investigate acceptable learning objectives, the potential of PM to relevant professions and motivation of participants. Quantitative investigations using rating scales and visual analogues were incorporated. The focus groups were audio recorded, transcribed by intelligent verbatim and translated to English. NVivo was used for data analysis and interpretation following a hybrid approach using the Framework Method and thematic analysis. The analytical framework, Implementation Science, was applied to organise and present research data.

**Results:**

Precision Medicine is considered a new topic area, largely unfamiliar to frontline healthcare professionals.. There was acceptance of a more precision approach to care among all participants with perceived positive implications for patients. Valuable insight was gathered on acceptable content and form for training. All participants expressed concerns on readiness within their professions which included an insufficient system infrastructure, a lack of time to attend needed training, a lack of clarity for use in practice and the time needed to build a support network.

**Conclusions:**

A precision approach to patient care is on the horizon for health care professionals not only in hospital settings but also at the community level. Our results conclude that an adaptable and flexible training programme in PM is timely, contextually relevant and conducive to the needs of targeted health professions for successful implementation. A training programme in PM will require support across sectors and stakeholders, supporting insurance models, educated patients and integrated care supported by innovative technology. Implementation Science outcomes are a useful strategy towards design of an effective training programme that can have measurable impact in practice.

**Supplementary Information:**

The online version contains supplementary material available at 10.1186/s12909-022-03613-2.

## Background

New strains on healthcare systems including access to trained healthcare professionals have prompted discourse on how healthcare delivery must change to meet demands of more complex diseases, new knowledge and skills and evolving technology [1]. Delivering the right approach, for the right patient at the right time, could lead to more effective, efficient health systems with better patient outcomes [2], an approach known as Precision Medicine (PM). PM considers individual needs, variability in genomes, the environment and lifestyle [3] for more precise diagnostics, treatment and management plans. The global health stage is calling for impending change to current practice-care delivery (Bernaert, 2021). The promise of PM offers more targeted care pathways for patients, built on foundations of interprofessional collaboration [4]. Scientific trends report positive patient outcomes and call for PM as the new standard for care across specialties as seen in oncology [5, 6], cardiology [7, 8], and respiratory medicine [9–11]. New research showing positive patient outcomes, eg precision nutrition for type 2 diabetes [12], expands the applicability of PM to community settings including social, environmental and lifestyle factors. Yet promise of this approach is dampened by critics questioning whether expectations of PM is outpacing clinical reality [13]. Healthcare professionals are not yet ready to implement a more genome-driven approach to patient care [14].

Targeted training programmes for Continued Professional Development (CPD) for PM is lacking. Existing programmes from other fields that could serve as a model are often tainted by design that is ad hoc, and top-down [15], with little follow-up or known impact in clinical practice [16]. Effective training targeting frontline healthcare professionals may provide one of the keys to unlocking the challenges of PM implementation. For education to be effective, it must be implemented well. Implementation Science suggests that a quality innovation does not guarantee uptake, and so facilitation of the transition from research to practice is essential [17]. The application of Implementation Science to the field of education is still relatively new [18], which serves to highlight important relevant findings of supported use of this approach to training programme design in PM. To determine whether a training programme is successful, there is a need to evaluate the training programme as well as implementation in practice; the outcomes and impact [19]. In the context of this project, the objective is to design an effective training programme for PM, and investigate the level of knowledge, motivation to participate, and perceived potential of PM for advanced practice nurses, pharmacists and general practitioners.

Tailored education interventions that lead to behaviour change in daily practice rely on needs assessment [20, 21]. A needs assessment has been described as a systematic approach to investigating the state of knowledge, ability, motivation, or attitude of a targeted audience towards a particular subject area [22]. The intended objective for this study was to begin the training programme design with a bottom up approach, to investigate needs of the target groups and the perceived gap between existing competencies and those required for a precision approach for clinical daily practice [23]. Acknowledgment of the essential role of assessing needs is not new, yet comprehensive and standardised tools are still lacking [24]. Relevant examples of needs assessment studies include approaches to investigate knowledge gap and barriers to implementation [25], content and form of training [25, 26], as well as level of interest [27].

This research describes the first steps taken towards development of an education intervention for of APNs, pharmacists and general practitioners to improve knowledge, skills and behaviours towards PM. The objective of this research project was to identify the current knowledge, content of training required, perceived potential and motivation to attend a PM training, among the targeted professions, investigated with the following research questions:

- Q1 What is the current level of knowledge in PM?
- Q2 What content and structure for PM training is acceptable?
- Q3 What is the perceived potential of PM?
- Q4 What is the motivation to attend a training programme in PM?

## Methods

### Study purpose and related frameworks

An exploratory approach was used as a methodological framework fitting to the education aims of this study [28]. The implementation of interprofessional focus groups was selected as an appropriate method to explore our research aims. Focus groups are deemed appropriate as a platform to share views and challenge opinions on experience [29]. In this article we report on a first exploratory phase, with the specific objective of gathering perspectives of the target audience relevant for training programme design. The use of focus group studies matched the study aims as this research addresses a poorly understood topic [30, 31].

This research was guided by two specific conceptual frameworks [32]. Kern’s instructional design model served as a basis to guide the problem identification and general needs assessment. This approach clearly outlines methods that include a review of literature, curriculum documents from other institutions, and clinical practice guidelines [33]. Four questions were identified to investigate the level of knowledge, the content and form for learning, perceived potential and motivation that find their roots in Kirkpatrick’s evaluation model. This model was used with the purpose to begin with the end in mind. In this research context, the approach taken to investigate needs as a first step in design is aligned to the steps required to consider implementation needs, to identify actions for implementation of the programme and potential determinants (barriers and facilitators) [34]. This research highlights the benefits of adopting implementation outcomes to better understand barriers, and outline targeted steps towards effective implementation. These frameworks guided an iterative process for data collection and analysis*.*

### Design

Two interprofessional focus groups were delivered in Bern (in German) and in Lausanne (in French), following the same design. Focus groups followed a prepared question route and was guided by activities including open-discussions, ranking of content, and group discussion all facilitated by a moderator (supplementary file 1). Consistency across both focus groups was maintained by ensuring that all supporting materials (charts, question route and group activities) were prepared in English and translated in German and French by the same (bilingual) researcher. Also 2 of 4 team members took part in both focus groups to maintain consistency. Participants received no content information about the research project before their participation.

### Participants and researchers

*Targeted participants* included 4 general practitioners, 4 APNs, and 4 pharmacists (*n* = 12) equally distributed across both focus groups. By design, it was decided not to include an additional participant per profession to each focus groups to ensure a flow of conversation and follow best practice in focus group size [35]. The participants were recruited by project leaders (Professors of internal medicine, general practitioner medicine and medical education). Purposeful sampling was applied [36]. Clinical experience and regular patient contact were also criteria for selection, as well as gender, and years’ experience.

*Researchers* included 4 physicians with a special interest in PM (JC, IG, GW, EJ), 3 medical educationalists (SG, FS, SM), and 1 psychology research student and registered nurse (EvK), and a project coordinator (PC). Members of the research team were assigned roles; a moderator (FS, EJ), a co-moderator (EJ, SM), a note-taker (EvK) and a content expert (EJ), following a best practice approach [30]. Practice sessions ensured all research team members became familiar with their roles, the content and techniques to encourage participants to share their views in a comfortable environment [37] and for consistency in data collection [38].

### Data collection methods

**Fig. 1:**
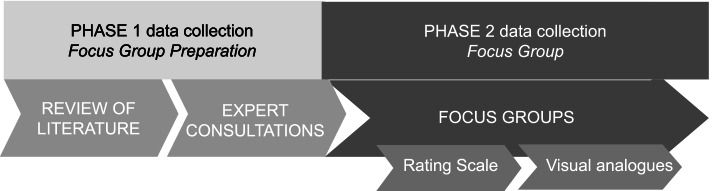
Data collection instruments presented in two phases

Specific instruments were used to gather data in two distinct phases. A review of the literature and expert consultations focused specifically on focus group preparation. The results of this phase were used as a foundation from which to further explore the research questions in phase 2 (Fig. 1). Rating scales and visual analogues were used to collect quantitative data during the focus groups.

#### Data collection—phase 1

A *literature review* [39] was used in a first step, to derive a definition of PM. PubMed, Medline, Google (also -Scholar and -books) were searched by a member of the research team with the following key terms: PM in Primary Care/ Personalised Medicine in Primary Care/ Genomics in Primary Care/ Pharmacogenomics. The researcher logged publications based on opinion of relevance, formal and informal qualitative and quantitative studies, literature reviews and year of publication (2014 to 2020), with the objective of seeking out key terms and descriptions of PM. The collated descriptions or definitions of PM were reviewed by members of the project team and descriptions deemed relevant were selected based on a recurrent theme and/or combination words or phrases that included genetic/genomic/environment/lifestyle/big data/assess risk/health outcomes/prevention interventions/treatment strategies.

A second *literature review* gathered sources on content for training presented in supplementary file 2. A list of learning objectives for a training programme in PM was assembled from educational resources and existing online training programmes in PM. These learning objectives targeted APNs, pharmacists and general practitioners.

*Consultations with experts* and reviews of external institutions curricula are consistent with methods used in general needs assessment [33]. From the content areas found from the review of the literature, topics were assembled into relevant learning objectives in an iterative consultation process with content experts and medical educationalists. This was used as a key reference document to explore acceptability of content during the focus groups.

To adequately explore the perceived potential of PM among participants, it was necessary to illustrate to participants how PM could be applied to their own professional roles, due to reported lack of knowledge of PM among health professionals. For that purpose, the research team prepared patient cases integrating a precision approach to care specific to APNs, pharmacists and general practitioners. These cases were developed by an internal medicine specialist and reviewed through iterative consultations with members of the research team (primary care specialists and medical educationalists). Cases were designed to take less than 10 min to read.

#### Data collection – phase 2

The findings from phase one data collection were used as the basis from which to build a question route for the focus group study.

The preparation of the *focus groups* followed a design structure purported by Nagle and Williams 2013 [31] outlining 5 distinct and separate stages (identify the study purpose, prepare the question route, prepare the focus groups, deliver the focus groups and analyse data) [31, 40]*.* The question route was designed to gather participant’s perceptions linked to the 4 research questions (Table 1).

**Table 1:** Focus group question route linked to the research question objectives

Investigation linked to the research questions	Question Route	
**Research Question 1 investigating the level of knowledge on PM**	**How would you describe Precision Medicine?** [Presentation of description of PM from literature] •How do you find this definition? Is it understandable, conclusive, concrete, and practical? Where are the parallels and where are the differences compared what you have found in your previous activity? •Overall, to what extent do you agree with the definition of PM?	
**Research question 2 Investigating acceptable content and a structure** **for training**	**Now that you know a bit more about precision medicine, what in your opinion are the knowledge and skills that would help you to practice a more precision approach?** [Rating scale with learning objectives] •Which learning objectives are central to successfully applying PM from the perspective of your professional group? •How do you prefer to learn? •What types of courses are most interesting for you?	
**Research Question 3, investigating the** **perceived potential** **of PM**	**If we imagine an era of precision medicine, what would be your role?** [Presentation of case studies based on professional roles] •How realistic do you think the roles are described [in the presented case studies]? •How much can you identify with the described role from your professional field? •Where do you see particular potential? What would you like to share?	
**Research Question 4, investigating** **motivation to attend a training on PM**	**What is the level of motivation for a training programme on Precision Medicine?** **[Activity using visual analogues]** •What is your level of motivation to attend a training programme in PM today •What is your level of motivation in a training programme in PM in the future (in 5 years)?	

The full question route and instruments for the focus groups are found in the supplementary files, and were piloted with two general practitioners, a pharmacist and an advanced practice nurse (supplementary file 1). Feedback from pilot participants helped the lead researchers to refine the question route.

Practice rehearsals of the focus groups were held in German and French with materials and props, following reported best practice [41]. This process tested how questions were understood, the approximate time needed and served as practice for the team. Items that were confusing or redundant were identified and improved. Participants who took part in the practice rehearsals were not included in the focus groups.

A *rating scale* was designed to allow participants to rate each learning objective from 1 (not at all important to my profession) to 10 (extremely important to my profession). Participants rated each learning objective*,* to gather data to investigate acceptable content for a training programme.

*Visual analogues* were prepared on charts and participants were asked to map their level of interest on two individual scales to investigate motivation [42]. *What is your level of motivation to attend a training programme in PM today* and *2.What is your level of motivation in a training programme in PM in the future (in 5 years)?* The presented questions aimed at exploring the motivation to engage in PM training among participants.

### Data collection and analysis

Data collection for phase 1 preparation of the focus groups took place between February and August 2020. Expert consultation meetings took place monthly during this time with all project members and the smaller research team conducted more regular weekly meetings (FS, SG, EJ, IG, SM, EvK). Due to Covid-19 Pandemic restrictions, only one face-to-face project team meeting was delivered in February 2020, with the objective to refine and agree on a description of PM.

Data collection for phase 2 focus groups took place in face-to-face meetings following pandemic safety guidelines in August and September 2020 at the University of Bern, and University Hospital Lausanne respectively. 6 participants with equal professional representation took part in each focus group [43]. Both focus groups were planned for 3 h and ran over time but not beyond 15 additional minutes. The focus groups activities and discussions were audio recorded. All visual results were photographed or collected onsite and used for the subsequent analysis. Recordings of the focus groups were transcribed in German and French, translated into English and passages of the text back-translated [44] to check translation quality. SM and PC discussed key concepts of specific passages, reaching final agreement on more ambiguous passages of the translations [45].

The data from both focus groups were analysed using the Framework method of analysis [46] and thematic analysis [47]. NVivo was used for data analysis [48]. After familiarizing with the transcripts, SM identified and organised codes and patterns within predetermined themes characteristic of the Framework method [46], related to the research questions. The Framework method of analysis guides data collection identifying a priori themes in a deductive top down approach. This method also recognises new data from guided discussion [46]. The inductive codes were generated by NVivo. Inductive and deductive codes were sorted by SM and potential sister themes to the original question route (deductive codes) or sub-themes to new or existing topic areas were presented (inductive codes). Conducive with the Framework Method to compare groups using matrices, SM investigated differences across professional groups and focus group settings. The codes were discussed in weekly meetings between January 2021 and March 2021, in which SM, EJ, FS, and SG refined codes. EJ blindly rated one full focus group for interrater reliability by investigator triangulation [49]. SM kept a visual coding logbook throughout the process, noting the decision trail to themes and an organising structure outlining rationale for decisions [50]. This resulted in a visual progression of results, with evolving themes and sub-themes that could be tracked and shared across the research team.

### Analytical frameworks

During the final analytical phase, outcomes of Implementation Science were found helpful to map key themes, and to understand the implications of the wide-ranging themes identified in analysis. Implementation outcomes situated within the scientific study of methods purported through Implementation Science, serve as indicators for success and in the design phase offers preconditions for attaining desired change [51]. An example of this approach has been successfully reported [34]. The Implementation Science framework visualises implementation of evidence-based interventions in real world settings and shows high relevance for the overall venture of this project. Relevant to our research are the constructs of acceptability, appropriateness and feasibility *Acceptability* describes the reaction of the target audience that a specific intervention, treatment or innovation is agreeable, palatable or satisfactory. Qualitative exploration of acceptability seeks to investigate focused questions on understanding of the specific topic [52]. *Appropriateness* is the perceived fit or relevance and is the construct that captures barriers to implementation. In the context of this study, the application of appropriateness systematically organises perceptions on the relevance of the topic to the professional roles [51]. *Feasibility* is considered the extent to which an innovative intervention can be successfully implemented within the targeted setting and considers the potential success or failure of an intervention [51].

### Ethical considerations

All focus group participants received both written and oral information and signed an informed consent form. The qualitative research was conducted after successful validation of our research application to the Swiss Cantonal Ethics Board in April 2020.

## Results

Results are presented from the data analysis as illustrated in Fig. 1.

### Results of phase 1

In the first literature review, 21 relevant articles were identified by the lead researcher.6 articles were identified as relevant and used to build statements on PM and presented to 2 primary care specialists, and 2 internal medicine specialists for validation [3, 14, 53–56]. The descriptions were edited and refined to the final agreed description of PM. This description was used as a baseline from which to ensure that all participants began the focus group with a common and agreed understanding of PM. The description assembled from the literature and consensus with experts was presented and discussed; PM is *an approach that takes into account individual preferences, variability in the genome, environment and lifestyle in order to tailor prevention interventions, diagnosis techniques and treatment strategies to improve health outcomes*. All participants agreed and accepted the presented definition of PM to launch further investigations into research question 1 on the level of knowledge in PM.

In a second review of the literature investigating acceptable content and proposed topic areas, 21 relevant citations were found as a result of the review of the literature (supplementary file 2). Through expert consultations with content experts and medical educationalists, the content found within these citations were iteratively developed into specific learning objectives. The result of this process led to a list of 7 learning objectives presented to focus group participants to investigate acceptability using a rating scale.

3 patient cases integrating a precision approach to care specific to APNs, pharmacists and general practitioners were developed through expert consultation by the research team. The cases were used to investigate the perceived relevance of PM to each professional role further investigated during the focus groups (supplementary file 3).

### Results of phase 2

Participants included 4 general practitioners, 4 APNs, and 4 pharmacists (*n* = 12) equally distributed across both focus groups. Of 12 participants, 6 were male & 6 female. Median age was 40 years (range 29–64). Mean years’ experience was 14.6 years with a range of 5 years to 42 years’ experience.

**Fig. 2:**
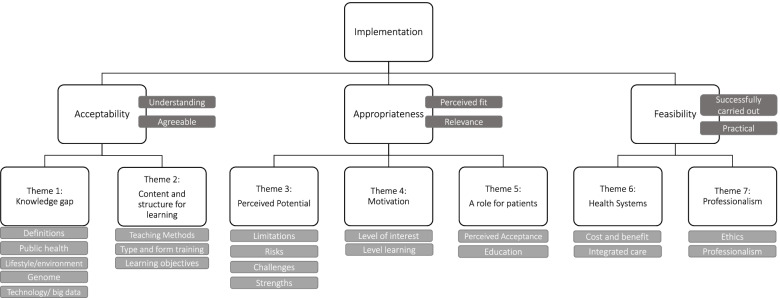
Resultant themes mapped to an analytical framework of Implementation Science outcomes

Figure*2* presents the resultant themes and subthemes organised within 3 overarching implementation outcomes of acceptability, appropriateness and feasibility. Deductive themes represent the objectives of the 4 research questions. Inductive themes emerged as Theme 5 a role for patients, Theme 6 health systems and Theme 7 professionalism. We report our findings following this structure. We recognise that each outcome has additional facets beyond what is presented in this figure [51, 52]. The iterative process applied to decide on final themes strengthens credibility, whereby themes determined by researchers appropriately fit focus group discussions [57]. Using NVivo, the research team investigated differences across professions and settings. No differences were found.

### Acceptability

The reported understanding of PM among the participants was low. At the onset of both focus groups, we found a general reluctance to support a training programme in PM. This could be attributed to limited knowledge on the topic and a lack of understanding of how training content could be applied in practice. As discussion progressed, participants engaged in discussions on PM and openly became more agreeable seen from the results of discussions on proposed content for training.

#### Level of knowledge – research question 1

The objective to address the first research question was to explore whether frontline health care professionals were knowledgeable about PM. For the context of this study the knowledge gap is referred to the lack of competence to implement a more precision approach to patient care. Half of all participants (*n* = 6) self-reported that they had not previously heard of PM. No participant reported a patient request for genomic sequencing. No participant reported having seen a patient that presented with genetic data.

There’s a huge gap, I’m starting from nothing

The practical application of genomic testing was discussed, e.g. when it was appropriate to request a genome sequence, how that information should be managed and shared with patients, raising questions on how best to translate genomic data to better patient outcomes. Participants reported a lack of knowledge on how they could apply PM in their own practice.

#### Content and structure for learning – research question 2

*The rating scale* investigated level of importance of each learning objective, from 1 (not at all important to my profession) to 10 (extremely important to my profession). There was high agreement on relevance of the presented learning objectives (supplementary file 4: mean agreements between 9.75 and 6.25) within each of the presented topic areas. Ethics, big data, and understanding of insurance models also emerged as potential topic areas, but not necessarily required for a targeted and foundational programme in PM.

An additional subtheme on the *type and form* of training emerged reflecting expectations in practice. Given the nature of PM as an integrated approach to care, participants supported interprofessional training;

*It would be an advantage to have a common training …as participants have to work together, so why not learn together… ….There might be a common foundation… …and we can address specific roles* [general practitioners, pharmacist, advanced practice nurse].

Flexible learning adaptable to each profession was found as a challenge. Attitudes towards types of learning online formats or onsite formats differed between individual participants. Our findings highlighted that every participant had individual expectations and preferences, underlying the importance of a design that is adaptable to every learner. Participants did recognise that the form of training must support the delivery of PM, where interprofessional collaboration is part of effective delivery of PM.

*It's the big challenge for online training sessions, to create interactivity between participants… you will not be able to do 100% online if you want to develop those particular skills in precision medicine.*

### Appropriateness

The challenges of implementing a precision approach to patient care were discussed extensively, whereby participants outlined numerous barriers, many of which are structural in current healthcare delivery, for example insurance models.

#### Perceived Potential – research question 3

##### Strengths

Participants discussed the potential of PM as an innovative approach to patient care that will open the door to new requests from patients and hospitals particularly around genomic sequencing and genetic information. Also the need to promote better integration and interprofessionalism across settings, as well as early intervention and better patient management.

*The professional organisation in which I am involved is generally favourable to progression towards PM and to the inclusion of PM in our logic. This organisational system would promote opening towards innovation in patient management.*

Participants agreed that the presented patient cases had high relevance to their profession. Noted limitations included a lack of vision on how to implement PM, lack of clarity on clinical outcomes for patients, and access to patient records.

‘*So for the pharmacist scenario……we don’t have access to patients’ medical records and we are fighting for that, because that would increase the safety with which we dispense medication’.*

*Risks* of implementing a PM approach that could potentially have a negative impact included big data, safe collection and storage of data, and the risk to patients.

At the level of accessibility of data …. We may end up with a massive amount of information and then we need to know how to interpret it….and make the right decisions.

*Challenges* were identified as a lack of time, lack of resources, how to share information, absence of a clear definition. Many of these challenges were repeatedly expressed throughout discussions.

We are actually overwhelmed with all the things we should do in this short consultation. 30 min is too short, it takes a whole hour for this kind of patient.

#### Motivation – research question 4

The results of the *visual analogue* were plotted to a 1–10 scale for each participant. The current perceived importance of a training programme in PM was ranked as low (indicated as an average of 1). However, all professional groups indicated an increased importance of PM training as well as increased level of interest in PM in the future (between 4.5 and 8.2). Investigating the intrinsic level of interest to attend a training programme in PM unveiled additional extrinsic factors that may hamper a genome driven approach to patient care. The level of motivation in PM today was low due to a reported lack of readiness from insurance, patients and health systems, lack of time, and the absence of PM from clinical guidelines, described as motivating factors by participants.

I would say …, that my degree of interest (for PM training) would depend ultimately to what extent PM enters into [clinical] guidelines.

#### A role for patients – inductive theme

The implementation of a more precision approach to patient care could not be successful without interest from patients.

If I didn’t have interest from patients…, I wouldn’t be interested.

Education in PM can equip patients to make life choices that may reduce risk of disease presentation.

It is not enough to have the right tool which gives the right intention, you have to help the person go in the right direction

An educated patient who is willing to have their genome sequenced and is open to discuss how to reduce and identify risks through lifestyle choices and preventive medication may benefit from an extended and healthy life.

### Feasibility

Following this qualitative evaluation, there are systemic challenges embedded within the current health care model that may facilitate or hamper implementation of a precision approach to patient care. Interestingly, PM was not seen as an ‘all or nothing’ possibility in this context. Participants discussed that PM will evolve over time. From these discussions we can deduce that the level of implementation will be dependent upon the structure of health care systems.

#### Health Systems – inductive theme

The participants understand PM as a care approach that is integrated through professional networks that facilitate a patient’s care transition across healthcare settings.

The more interprofessional interaction there is, the better the integration….by managing as a network we integrate more.

Integrated care is dependent on interprofessional practice, availability of patient data across settings, and communication channels.

Around costs, two diverging perspectives emerged; the additional costs of genetic testing and support treatments, and the delivery of more efficient medicine that may yield better patient outcomes. The potential of implementing an individualised approach will be restricted by current infrastructure, particularly with patient health insurance. In a number of scenarios PM requires support of many healthcare professionals.

If it leads to the consultation of a dietician [for example] if I look at today’s reality, the patient costs will not be covered.

#### Professionalism – inductive theme

The risks of implementing a more genome centred approach surfaced discussions on the need for clear guidance to safeguard patients. In this instance, the collection, storing and disseminating patient data, risk of quality of life to patients and safe decision making.

In the end is there not a risk of selecting individuals who deserve to be treated based on their genome? … [Precision Medicine]… should be implemented ethically and to the highest standards.

The movement of professional boundaries is evolving in current delivery of care to patients. Professional roles are not always clearly defined and sometimes dictated by legal restrictions rather than capabilities of professionals or what makes sense in practice.

The pharmacists are currently redefining their profession and the neighbourhood pharmacist is a gateway to the healthcare system.

Participants demonstrated a commitment to delivering the best care to patients through professional identity and ownership of tasks.

*In interprofessional work there are two dimensions which are important. One is to work with the other person… the other is to fully understand one's own identity and be able to communicate it to others.*

## Discussion

Following this qualitative evaluation, delivery of a training programme will be challenging with low levels of knowledge about PM, doubts on the relevance to participants’ current professional roles, and the belief that health systems are not ready to implement a more precision approach to care. Following discussions in the focus group, participants’ acceptance towards PM became more open with specific examples of how PM is relevant to them in their daily roles, and the types of foundational objectives that will be required to raise the knowledge of PM among frontline healthcare professionals. Use of genomic-guided precision medicine to support targeted prevention, diagnosis, treatment and management of patients must be prioritised to meet current and future health needs [58]. Reflecting on results of this project, targeted education can support to break through these barriers and facilitate the delivery of PM in order to reach its full potential.

### Acceptability

If targeted professions do not understand PM, it is unlikely that PM will be deemed an acceptable approach to care. This finding is indicative of previous research stating that the acceptability of any intervention is likely to increase with increased knowledge of that intervention [59]. Providing participants with specific practical examples of PM to their relevant professions opened the door to insightful discussions on specific learning objectives relevant to their role.

#### Level of knowledge – research question 1

Participants were unfamiliar with the application of PM in practice. The PM knowledge gap among healthcare professionals has been explored as a potential barrier to implementation [60]. There is perceived limited evidence of clinical usefulness and limited understanding, congruent with previous research in Primary Care [56]. Our findings support the need to design a training that increases knowledge of PM with practical examples for use by targeted professional roles.

#### Content and structure for learning – research question 2

We found an acceptance of proposed learning objectives across professions responding to *research question 2* to assess acceptability of content for training. The results shared in *supplementary 4* illustrate the overall high level of perceived importance across professions. A key focus in the development of learning objectives was the practical application of PM, which may offer an explanation of these results. A few additional topic areas were discussed, including big data and data protection, ethics and translating PM to patients which will be considered as content areas in training programme development.

Interprofessional learning was discussed as the preferred approach, with implications for content; *‘as participants have to work together, so why not learn together*’. Consistent with findings in the literature, practical implementation relies on interprofessional education that will support communication across settings in a robust integrated care delivery [61]. Implementation of a training programme in PM that is fit for purpose for each professional group presents inherent challenges to design. Examples of inter-professional flexible learning programmes using a modular approach and sequential learning are successfully published [62, 63]. It is intended in the design of this training programme to clearly indicate module objectives and the relevant target population for each module. Use of a modular approach in PM training will offer a flexible structure to build a learning pathway that can guide learning specific to the targeted professional group. A potential critique of our findings indicates that a favour towards interprofessional learning is an effect of the interprofessional mix of the groups, rather than a real need.

Online and blended learning approaches are deemed most favourable to meet the demands of busy healthcare professionals [25]. In our study, this is exemplified by the participants need for online learning, due to resource and time constraints, and recognition that onsite training would be required to bring more inter-professional aspects demanded by the topic of PM, to a training programme.

Further evidence is needed to determine what teaching methods are effective to bring measurable impact and improved care to patients in clinical practice [64]. Our findings add to a call for more flexible, adaptable and a blended approach to learning in CPD [65]. This needs assessment has enabled designers to test the waters with a structure and content for training in a topic largely unknown to frontline healthcare professionals. The intended next steps will be to present a more refined training programme to wider targeted professions for validation.

### Appropriateness

A fit for purpose training programme requires consideration of the perceived fit and relevance and in this context how PM could translate to current everyday practice for our targeted professionals. Investigating appropriateness according to Implementation Science theory, is to identify the barriers to implementation so that targeted solutions can be built into design [51]. We identified specific barriers to implementation, for example the need for pharmacists to access full patient data is not currently possible. We deduce from findings that integration of a PM approach will require specific milestones and realistic expectations of what can be delivered within the constraints of today’s health systems, to a more integrated approach to precision patient care that we strive to deliver in the future.

#### Perceived potential – research question 3

Participants viewed PM as an innovative approach to care, which also aligns to previously published articles on healthcare innovation through PM [66], shedding light on the research objective to gather insight on the perceived potential of PM to the targeted professions. Obstacles to introducing innovation in patient care are not new, and therefore should also be considered within the context of this project. The presentation of pre-prepared patient cases (supplementary file 3) was crucial to opening discussions linked to practical and realistic scenarios bound by restraints of the current health system. Our findings add to previously published research outlining limitations of implementing PM including, unprecedented data volumes, interpreting data at the level of the individual and integration into the clinic [67]. As seen from this research, specific challenges will include additional demands from patients, particularly requests for genomic sequencing, the need for integration across health care settings, and the sharing of data across health settings.

For innovative approaches to care, additional caution is required to overcome inherent challenges in design and implementation [68]. Covering these topics within a targeted training programme will equip frontline healthcare professionals with the know-how and skills to overcome barriers and maximise the potential of PM until this approach evolves past infancy and healthcare systems can fully support implementation.

#### Motivation – research question 4

Results of this exploration align to the motivational model described by McMillan, McConnell and O’Sullivan 2016 [69]. Interest was explored as the intrinsic motivationof our target audience to attend a training programme in PM [70]. Our findings conclude thatthe targeted professions will attend a training Programme in PM not only when interested, but also when motivated by external consequences. Extrinsic factors are essential to drive motivated behaviour [70], particularly in this unfamiliar field of PM. These findings are aligned to the nature of motivation reported by Ryan & Deci [70]. Extrinsic motivating factors in this study were reported as when PM enters into clinical guidelines, safe management of patient data across settings and the need to change insurance models, as examples. Targeted clinical guidelines based on efficacy and effectiveness of PM in practice can provide one of the keys to unlocking successful implementation of a precision approach to patient care [61]. From these findings we deduce that attendance, as a critical success factor, is driven by motivation.

#### A role for patients – inductive theme

This theme emerged from new inductive findings not established as a research objective. Our conclusions open the door to essential considerations to educate patients and build awareness in PM. ‘*It’s not enough to have the right tool for the right intention, you have to support that person to go in the right direction’*. A reported lack of understanding and awareness of PM among patients and public continues to be reported [71]. New studies are presenting refreshing insights that most patients are willing to share their data and biospecimens for research [72], and so accepting PM as an approach in practice. These findings echo the growing role of patient involvement as a catalyst for change [73]. For PM to *fit* to clinical practice, awareness and education are essential to train patients to absorb complex information about their health to enable them to make informed choices [74]. Additional interventions to increase knowledge about PM among patients will support the overall impact of this training.

### Feasibility

From these targeted discussions, the success or failure of a precision approach to patient care was deemed an indicator for the success of failure of delivering a training programme in PM. This section of results emerged as key findings from open discussions during the focus groups, and outside of the identified research questions, yet crucial to the success or failure to training with impact. If structurally, a health system is not prepared to deliver PM, then it is unlikely a training programme in PM will be effective. The essential reflection of participants on the presented cases during the focus group is that implementation of PM will be an evolution to a new approach to patient care.

#### Health systems – inductive theme

Participants identified the role of interprofessional practice as a prerequisite to precision patient care, describing examples of a PM-friendly care system that strengthens communication, shares patient data safely across settings, and facilitates a network of experts. Participants agreed that building a network of professionals can mobilize multi-professional teams to foster integration across healthcare settings. These findings are consistent with effective patient interventions that rely on integrated care across clinical settings [75]. PM cannot expect to be integrated into insurance models without proof of cost-efficiency. These issues support a recently published report questioning whether PM facilitates better healthcare [76]. This report is contextually relevant for the Swiss system and calls for the need to reform insurance payment systems, making patients responsible and PM affordable. It offers potential solutions to an economic infrastructure already being considered by insurance, pharmaceutical, academic research, regulators and patients. *Precision Medicine: A Global Action Plan for Impact*, reports on national strategic implementation plans across 17 countries, including considerations for economic impact analysis [77]. Relevance of findings are not only limited to Switzerland. Similar investigative efforts on readiness, economic evaluations and partnerships to drive support technology from multiple stakeholder perspectives, are being reported world over [55, 78].

#### Professionalism – inductive theme

PM requires the highest quality professionalism in a coordinated effort across professions. According to participants, the future of PM could fly or fail based on the desire of professionals to move beyond current practice, take ownership for tasks (that should be revisited within professional roles) with a strong professional identity that supports practice precision for patients. The role of professionalism will require facilitation and empowerment of professions based on capability rather than restrictions imposed for legal or financial implications. Further research may shed light on the importance of professional identity as a success factor to implementation. Ineffectiveness in delivery brings risks to patients as well as ethical implications [79]. We have documented challenges resonating with participant’s reports on the need to safely store and disseminate patient data, facilitate safe decision making and offer accessibility to all patients without barriers. Safeguarding patients from the risks of privacy and discrimination [80], empowerment to make decisions [81] and access [82] requires thoughtful consideration of a new approach to care that is patient centred.

### Study limitations

Qualitative research has limitations of objectivising facts and more focus groups could have been run to substantiate themes. Our objective was to provide insights on reactions and potential for PM, proposed content and structure for CPD and motivation to adopt a PM approach. Despite the sample size of 2 focus groups, insights illustrated synergetic results and met the research objective with the intention to deliver a follow up survey targeting a larger population to substantiate findings.

Needs assessment must be practical. The research team continued development of a training programme during the results analysis and continually informed content development. This project simultaneously continued to conduct investigations alongside project development which should lead to continuous improvement towards a blended and inter-professional training programme in PM.

The research scope has limited investigations to targeted professions. Further stakeholders including patients, policy makers, educators, and health economic experts will need to be involved for PM to reach its potential as an accepted approach to patient care. At the onset of this study, the team focused on a study design to investigate the current knowledge of PM, the perceived potential of PM, acceptance towards presented content and a structure for training, and intrinsic motivations. Reflections on results add to convincing arguments for the practical application of *feasibility* as a specific outcome of Implementation Science in education design [83].

## Conclusion

This project set out to collect insights to inform design of an effective training programme in PM targeting APNs, pharmacists and general practitioners. To our knowledge this is the first reported evidence based design for an inter-professional training programme in PM. Awareness of the needs of this target group is essential, and maximising the impact of our findings will require a team effort to translate these conclusions to actionable measures to deliver an optimal training in PM. A structured approach to needs assessment has proven effective to these project expectations leading to more insightful findings of an acceptable, appropriate and feasible solution for design guided by an approach to Implementation Science.

A qualitative exploration and analysis of the perceived needs of targeted professions has important implications for intervention design and informing future implementation strategies to improve a training programme in PM. Investigating the *level of knowledge in* PM supports initial assumptions that the topic is not well understood. An adaptable training programme that is flexible to individual needs is favoured to drive *learning*. The *potential* of the programme should be driven by the perceived fit and relevance to practice. Engagement is determined by specific *motivating factors* that can support *implementation* including strengthening interprofessional learning, integrated care across settings, safeguarding patients and patient data, improving awareness and education in the topic, continuing evidence based research and revising economic barriers and insurance models. A design of a practical training underlining the foundations of PM and case examples of how this approach translates to clinical practice was deemed most useful and favourable. These findings deliver insights for how to deliver an effective education programme in PM. Although this study took place in Switzerland, outcomes appear relevant beyond these national borders. The promise of precision frontline healthcare will rest on a number of crucial factors, of which targeted education remains essential.

## Supplementary Information

**Additional file 1.** **Supplementary File 1. **Research questionroute.** Supplementary File 2. **References used todevelop the proposed learning objectives. **Supplementary File 3. **Patient cases. **Supplementary File 4*. ***List of learningobjectives discussed, and ranked on a charted scale from 1 (not relevant) to 10(extremely relevant). 

## Data Availability

The dataset(s) supporting the conclusions of this article is available from the author on request by emailing the corresponding author.

## Abbreviations

CPD: Continuing professional development
PM: Precision Medicine
SEM: Standard Error of the Mean
F/G: French/German
