# Supplementary material for: Investigating acceptability of a training programme in precision medicine for frontline healthcare professionals: a mixed methods study
Source: BMC Med Educ. 2022 Jul 19;22:556. doi: 10.1186/s12909-022-03613-2 (PMC9294840; doi:10.1186/s12909-022-03613-2)
Supplement: Supplementary file 1 — Additional file 1. Supplementary File 1. Research questionroute. Supplementary File 2. References used todevelop the proposed learning objectives. Supplementary File 3. Patient cases. Supplementary File 4. List of learningobjectives discussed, and ranked on a charted scale from 1 (not relevant) to 10(extremely relevant). [file 12909_2022_3613_MOESM1_ESM.docx]

# Supplementary Materials

***Supplementary File 1: Research Question Route***

| **RESEARCH QUESTION 1: What is the current knowledge gap in Precision Medicine among the target professions?**  **Key Question: How would you describe Precision Medicine?**   The objective of this question is to assess the degree to which there is agreement with the presented description, and identify a if there is a perceived knowledge gap.  *1/4 Small Group Activity: Discussions in groups of two*   1. Designate one person per group to say in exactly 4 minutes* EVERYTHING you associate with PM. The other person writes it down 2. The roles of speaker and recorder are reversed (new time limit: 2 minutes). 3. Discuss findings and agree on a relevant content (5 minutes) 4. Write agreed items on post-its (2-3 minutes). Important: Please only 1 item per post-it! 5. Plenary discussion   *2/4 Expert - Input:*  *3/4 Presenting a description from the literature: How do you find this definition? Is it understandable, conclusive, concrete, and practical? Where are the parallels and where are the differences compared what you have found in your previous activity?*  *4/4 Plenary Activity: Overall, to what extent do you agree with the definition of PM?* |
| --- |
| **RESEARCH QUESTION 2:**  **What content and structure for Precision Medicine training is acceptable to target professions?**  **Key Question: Now that you know a bit more about precision medicine, what in your opinion are the key knowledge and skills that would help you to practice a more precision approach?**   The objective of this question will gather more information on the perceived level of importance of presented learning objectives by profession and to understand if some topics/competencies are more important than others.  *1/2 Single Activity:*  Activity - Which learning objectives are central to successfully applying PM from the perspective of your professional group? You will receive a list of 10 competency-based learning objectives. Please read through the list. Please rate each learning objective in terms of its relevance (cf. main question above) along the following scale: 1=not relevant at all for my occupational group to 10=extremely relevant for my occupational group. please rate self-added objectives along the scale. *What additional learning objectives could be added to the list?;* Short plenary.  If you cannot assess a learning objective, please tick 'NA' in the answer options.  *2/2 Brainstorming Activity:*  *Probe questions to support discussion example: Which types / methods of structured learning are most interesting for you?* (Lecture, seminar,...).  Just shout your answers out loud - we will record it on flipcharts! (2-3')  The plenary will reflect on which approaches are suitable for the project and could best pick you up and motivate you. |
| **RESEARCH QUESTION 3: What is the perceived potential of Precision Medicine among the target professions?**  **Key Question: If we imagine an era of precision medicine, what would be your role?**  The objective of this question seeks to assess the degree to which each of the participants accept Precision Medicine as a real possibility for the future of healthcare delivery.  *1/3 Single Activity: Presentation of cases.*  Please read each scenario carefully. Please ask yourself the following questions: *How realistic do you think the roles described are?* (Try to give reasons for your judgment).  Independent of your answer to question (i): *How much can you identify with the described role from your professional field? Where do you see particular potential? What would you like to share?*  *2/3 Small Group Activity:*  Form a group of three with one representative from each professional group.  Discuss a PM case in the group from the perspectives of your role with the other group members (15')  Describe your role based on the main task(s) from (2) on the role-specific flipchart provided (5')  Plenary discussion (5')  *3/3 Plenum Activity:*  *How high do you estimate the potential of the described roles, and impacts on patients?* |
| **RESEARCH QUESTION 4: What is the level of interest and motivation to attend a training programme in Precision Medicine?**  **Key question: What is the level of motivation to attend a training programme in Precision Medicine?**   The objective of this questions seeks to gather some insight from participant on the willingness/motivation to attend a training programme in Precision Medicine, or not!  *Question A: How interested are you now in a learning offer on PM?*  *Question B: How likely is it that you would take up such an offer in the near future (within 5 years)?* |

***Supplementary File 2: References used to develop the proposed learning objectives***

| 1. NIH - National Human Genome Research Institute. Introduction to genomics. https://genomicseducation.net/competency/physician  2. NIH - National Human Genome Research Institute. Provider Genomics Education Resources https://genomicseducation.net/competency/pharmacist  3. Biberstein, H. F. Médecine personnalisée. Bases pour la formation interprofessionnelle prégraduée, postgraduée et continue des professionnels de la santé. Recommandations 2019 de l’Accadémie Suisse des Sciences Médicales https://www.assm.ch/fr/Publications/Recommandations.html (2019).  4. Houwink, E. J. et al. Genetic educational needs and the role of genetics in primary care: a focus group study with multiple perspectives. BMC Fam. Pract. 12, 5 (2011).  5. Ginsburg, G., Willard, H. & David, S. Genomic and Precision Medicine 3rd Edition Primary Care. (2017).  6. NHS - Genomics England, Genomics Education Programme. Genomics in Primary Care https://www.genomicseducation.hee.nhs.uk/genomics-in-healthcare/genomics-in-primary-care/#toggle-id-3.  7. Skirton, H., Lewis, C., Kent, A., Coviello, D. A. & Members of Eurogentest Unit 6 and ESHG Education Committee. Genetic education and the challenge of genomic medicine: development of core competences to support preparation of health professionals in Europe. Eur. J. Hum. Genet. EJHG 18, 972–977 (2010).  8. Tognetto, A., Michelazzo, M. B., Ricciardi, W., Federici, A. & Boccia, S. Core competencies in genetics for healthcare professionals: results from a literature review and a Delphi method. BMC Med. Educ. 19, 19 (2019).  9. Paneque, M. et al. Implementing genetic education in primary care: the Gen-Equip programme. J. Community Genet. 8, 147–150 (2017).  10. Guttmacher, A. E., Porteous, M. E. & McInerney, J. D. Educating health-care professionals about genetics and genomics. Nat. Rev. Genet. 8, 151–157 (2007). | 11. U.S. National Library of Medicine. Genetics Home Reference, Your guide to understanding genetic conditions https://ghr.nlm.nih.gov/primer.  12. Burke, S. et al. Developing a curriculum statement based on clinical practice: genetics in primary care. Br. J. Gen. Pract. J. R. Coll. Gen. Pract. 59, 99–103 (2009).  13. Korf, B. R. et al. Framework for development of physician competencies in genomic medicine: report of the Competencies Working Group of the Inter-Society Coordinating Committee for Physician Education in Genomics. Genet. Med. Off. J. Am. Coll. Med. Genet. 16, 804–809 (2014).  14. Burke, S., Martyn, M., Thomas, H. & Farndon, P. The development of core learning outcomes relevant to clinical practice: identifying priority areas for genetics education for non-genetics specialist registrars. Clin. Med. Lond. Engl. 9, 49–52 (2009).  15. University of Dundee. Teaching and assessment for Health Data Science for Applied Precision Medicine MSc. Teaching and assessment https://www.dundee.ac.uk/postgraduate/health-data-science-applied-precision-medicine/teaching-and-assessment.  16. Talwar, D., Tseng, T.-S., Foster, M., Xu, L. & Chen, L.-S. Genetics/genomics education for nongenetic health professionals: a systematic literature review. Genet. Med. Off. J. Am. Coll. Med. Genet. 19, 725–732 (2017).  17. University of Glasgow. PRECISION MEDICINE (WITH SPECIALISMS) MSc. POSTGRADUATE TAUGHT https://www.gla.ac.uk/postgraduate/taught/precisionmedicinewithspecialisms/#whythisprogramme,programmestructure (2020).  18. American Medical Association. Precision Medicine and Genomics CME: Understanding Implications and Applications. AMA Education Hub https://edhub.ama-assn.org/pages/genomics-cme-course (2020).  19. Mikat-Stevens, N. A., Larson, I. A. & Tarini, B. A. Primary-care providers’ perceived barriers to integration of genetics services: a systematic review of the literature. Genet. Med. Off. J. Am. Coll. Med. Genet. 17, 169–176 (2015).  20. Tonkin, E. T., Skirton, H. & Kirk, M. The first competency based framework in genetics/genomics specifically for midwifery education and practice. Nurse Educ. Pract. 33, 133–140 (2018).  21. NIH - National Human Genome Research Institute. G2C2 - Genetics/Genomics Competency Center https://genomicseducation.net. |
| --- | --- |

***Supplementary File 3: Patient Cases***

## Patient Case 1: what polygenic risk scores could add to cardiovascular prevention

### Consultation at the GPs’ office.

A 48 years old male patient of Swiss origin comes to your consultation because he is very concerned about his risk of suffering a cardiac event. His father had a first heart attack at the age of 56 and his mother is diabetic. He already came to your office one year ago to know more about his personal risk. Besides asking about his family history of cardiovascular disease you measured the following variables;

- Waist circumference 101 cm
- Blood Pressure 125/83 mmHg
- LDL 2.8 mmol/l
- HDL 1.4 mmol/l
- TG 1.0 mmol/l
- Blood glucose 5.4 mmol/l

You also calculated and reviewed with him his 10 years risk of suffering a cardiac event (figure S1).


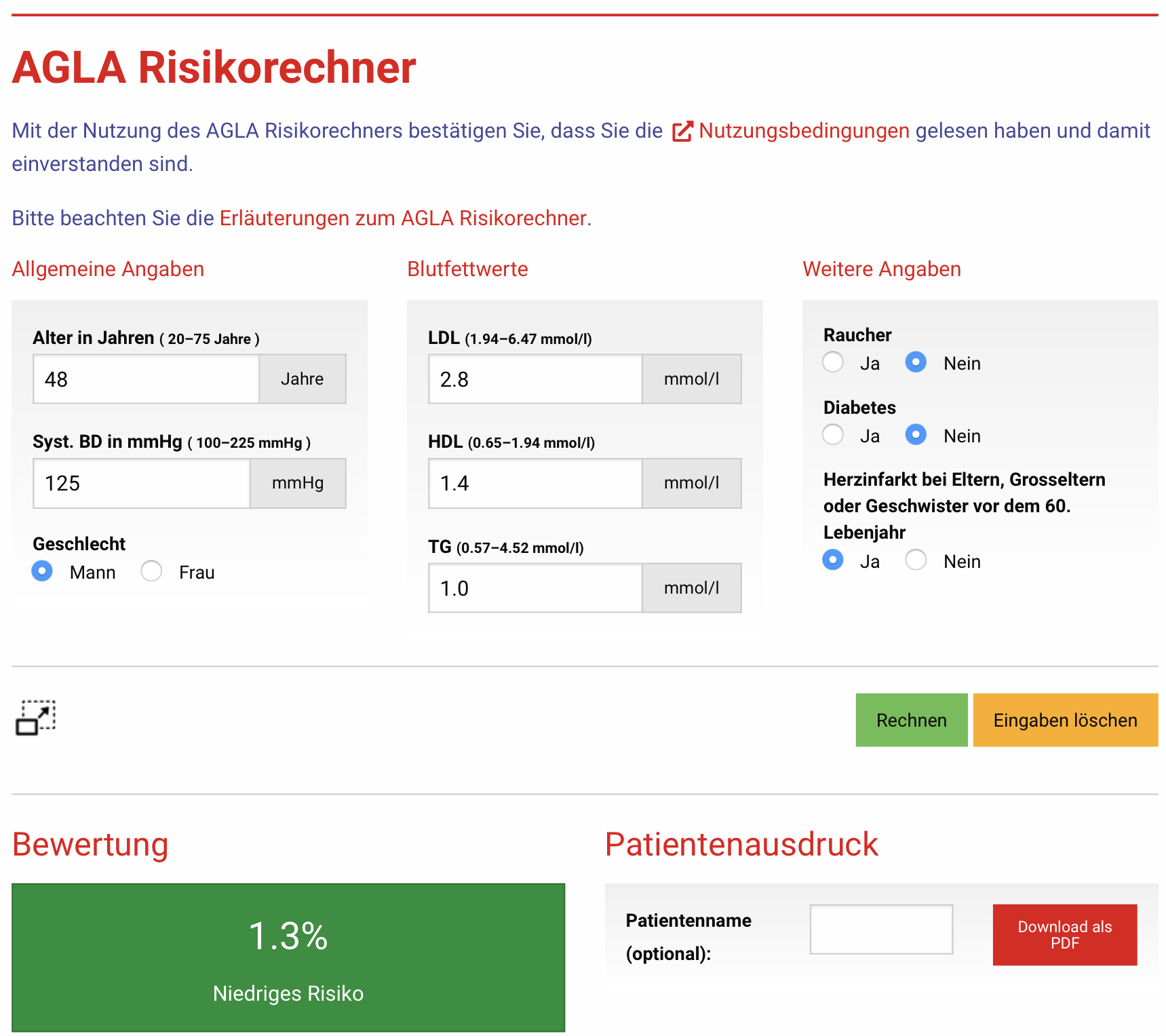


*Figure S1: the risk calculator*

*https://www.agla.ch/de/rechner-und-tools/agla-risikorechner*

The risk calculation reassured both of you and you advised to resume physical activity and to meet again in one year.

One year later, the patient comes back to you with a report from a research project linked to the local institutional biobank in which he took part and that calculated a cardiovascular polygenic risk score. He tells you that he has been invited by a multidisciplinary team to review this report but stresses that he decided to first review it with you. The report states that according to the polygenic risk score and compared to the general population, he has a 5.5 times increased risk of suffering a cardiovascular event. You spend time explaining what this information adds to his follow-up. You then measure his blood pressure and make a new appointment to draw blood in order to measure cholesterol and blood glucose levels again. He also tells you that he has not resumed any physical activity yet. You explain why it is paramount to resume physical activity and agree on an appointment with an advanced nurse practitioner specialized in genetics and genomics that is part of your network. You stress that one of his/her roles could be to help implement lifestyle changes.

### Consultation at the advanced nurse practitioner's office.

You review with the patient the differences between monogenic and polygenic illnesses with the help of specific iconography and stress the limitations of polygenic risk scores. You also emphasize the importance of environmental factors and refer to other studies that show that genetic risk can be partly reversed by adaptation of lifestyle. You counsel the patient on how to resume physical activity. You also propose to consult a dietician to improve the diet and lose weight and you both agree on setting goals to achieve. You schedule a new appointment in 6 months to monitor lifestyle changes.

## Patient Case 2: of medication and diseases: some don't match.

### **Consultation at the pharmacy’s office.**

19 years old adopted women of Cambogia Origin presents at your pharmacy with a medical prescription of nitrofurantoin obtained from a local emergency department for urinary tract infection. When you enter the drug name in the computerized prescriber order-entry system with built-in clinical decision support application, you get a warning that Nitrofurantoin is contraindicated in patients with G6PD deficiency (figure 3). The decision aid, also advises not to prescribe Sulfamethoxazole, Trimethoprim instead of Nitrofurantoin. After discussing with the Emergency Departement physician on the phone and again with the patient, you all agree to replace Nitrofurantoine by Fosfomycine. After further discussion with the patient to better understand her condition you propose to schedule a consultation with an advanced nurse practitioner specialized in genetics and genomics that is part of your pharmacy’s network. The patient accepts.

### **Consultation at the advanced nurse practitioner's office.**

### At your consultation, the patient explains that she purchased a Health and Ancestry service from the direct-to-consumer genetic service 23 and me one year ago with the hope of identifying her biological parents. She had loaded the report received by 23 and me into her electronic patient file but had bearly read the part of the report concerning health issues. You ask about consumption of broad beans and recommend their eviction. You also advise seeking medical consultation in case of fever/infection (figure S2). You explain the limitations of direct-to-consumer genetic testing and advise to confirm these results by performing clinical-grade sequencing of the gene coding for G6PD. You both agree that an appointment with a medical geneticist is important.


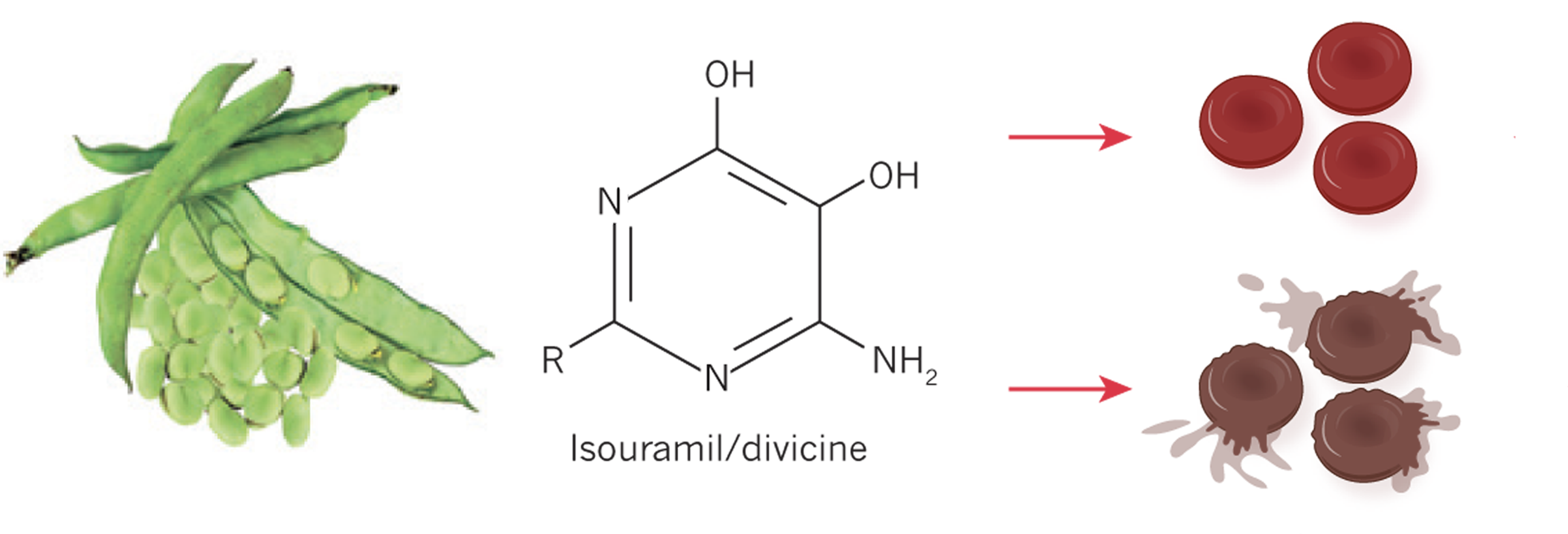


Broad beans

Wild type

G6PD deficient

*Figure S2: hemolysis by disruption of red cell wall*

*Broad beans are rich in Isouramil/divicine, which are highly oxidative chemicals. The red blood cells of people with G6PD deficiency lack anti-oxidant defence and these people are prone to develop haemolytic anaemia. Triggers of haemolysis are infections, certain medication and some foods.*

***Supplementary File 4:*** List of learning objectives discussed, and ranked on a charted scale from 1 (not relevant) to 10 (extremely relevant)

| **Acceptance of PM learning objectives by professional role** | | | |
| --- | --- | --- | --- |
|  | **Nurses** | **Pharmacists** | **Physicians** |
| 1] Use basic genetics knowledge and family history to integrate genomic risk into the health care plan^$^ | 9.75 ± 0.25 | 6.75 ± 1.00 | 8.00 ± 1.50 |
| 2] Understand, communicate and act on the possibilities, limitations and challenges of PM when integrated in the health care plan^$^ | 9.75 ± 0.25 | 8.00 ± 1.50 | 8.50 ± 1.25 |
| 3] Understand genomic sequencing information and consider/highlight its potential impact on health when counselling patients^$^ | 9.25 ± 0.50 | 8.25 ± 0.75 | 8.00 ± 1.25 |
| 4] Understand the relative contribution of behavioural, social, and environmental factors in health and disease and act on them to improve the health status of your patient^$^ | 9.75 ± 0.25 | 7.75 ± 1.50 | 8.25 ± 1.25 |
| 5] Understand and apply societal and legal implications of PM^$^ | 9.75 ± 0.25 | 8.00 ± 1.25 | 8.00 ± 1.25 |
| 6] Understand the clinical features and the therapeutic options associated with the most frequently encountered diseases of your specialty that are caused by genomic variants^$^ | 8.75 ± 0.75 | 9.50 ± 0.50 | 8.75 ± 0.75 |
| 7] Manage/coordinate care with PM services^$^ | 9.00 ± 0.50 | 8.00 ± 1.25 | 6.25 ± 1.15 |
| ^$^ Results are mean ± SEM. Elsewhere, results are numbers (%)  Green : ≥ 50 % think the sub-LO is rather useful. Orange : 50% think the sub-LO is rather useful but all participants are from the same regional focus group. Red : < 50% think the sub-LO is useful.  Rating Scale 1= not at all important to my profession 10= Extremely important to my profession | | | |
